# Comparison of the sensitivity of culture, PCR and quantitative real-time PCR for the detection of *Pseudomonas aeruginosa *in sputum of cystic fibrosis patients

**DOI:** 10.1186/1471-2180-9-244

**Published:** 2009-11-29

**Authors:** Pieter Deschaght, Thierry De Baere, Leen Van Simaey, Sabine Van daele, Frans De Baets, Daniel De Vos, Jean-Paul Pirnay, Mario Vaneechoutte

**Affiliations:** 1 Laboratory for Bacteriology Research (LBR), Ghent University Hospital, University of Ghent, Ghent, Belgium; 2 Cystic Fibrosis Centre, Department Pediatric Pulmonology, Ghent University Hospital, University of Ghent, Ghent, Belgium; 3 Laboratory for Molecular and Cellular Technology (LabMCT), Burn Wound Center, Queen Astrid Military Hospital, Brussels, Belgium

## Abstract

**Background:**

*Pseudomonas aeruginosa *is the major pathogen involved in the decline of lung function in cystic fibrosis (CF) patients. Early aggressive antibiotic therapy has been shown to be effective in preventing chronic colonization. Therefore, early detection is important and sensitive detection methods are warranted. In this study, we used a dilution series of *P. aeruginosa *positive sputa, diluted in a pool of *P. aeruginosa *negative sputa, all from CF patients - to mimick as closely as possible the sputa sent to routine laboratories - to compare the sensitivity of three culture techniques versus that of two conventional PCR formats and four real-time PCR formats, each targeting the *P. aeruginosa oprL *gene. In addition, we compared five DNA-extraction protocols.

**Results:**

In our hands, all three culture methods and the bioMérieux easyMAG Nuclisens protocol Generic 2.0.1, preceded by proteinase K pretreatment and followed by any of the 3 real-time PCR formats with probes were most sensitive and able to detect *P. aeruginosa *up to 50 cfu/ml, i.e. the theoretical minimum of one cell per PCR mixture, when taking into account the volumes used in this study of sample for DNA-extraction, of DNA-elution and of DNA-extract in the PCR mixture.

**Conclusion:**

In this study, no difference in sensitivity could be found for the detection of *P. aeruginosa *from sputum between microbiological culture and optimized DNA-extraction and real-time PCR. The results also indicate the importance of the optimization of the DNA-extraction protocol and the PCR format.

## Background

Patients with cystic fibrosis (CF), an autosomal recessively inherited disease caused by a mutation in the Cystic Fibrosis Transmembrane Conductance Regulator (CFTR) gene, are particularly susceptible to pulmonary infections with *Pseudomonas aeruginosa *[1,2]. Colonization of the airways of CF patients with *P. aeruginosa *results in higher morbidity and mortality because of the faster decline of the lung function, especially from the chronic infection phase onwards [3-5]. Detection of colonization and infection by this pathogen as early as possible enables to postpone the chronic infective stage and eventually to achieve the eradication of *P. aeruginosa *through early treatment. Indeed, early aggressive antibiotic therapy is now generally accepted as an efficient means to postpone chronic colonization [6,7].

In most routine laboratories detection of bacterial species in respiratory samples is achieved by culture. However, it has been shown that routine culture of sputa from CF patients yields limited microbiological information since it frequently fails to identify the pathogens, which were shown to be present by means of PCR [8]. Furthermore, the correct detection and identification of *P. aeruginosa*, although in general not a fastidious organism, is not as straightforward as frequently assumed [9,10]. To circumvent culture associated limitations, several molecular assays for the detection of *Pseudomonas *species have been described [8,11-19], Döring and colleagues [20] correctly remarked that, because of the influence of sample pretreatment, DNA-extraction protocol and the PCR format, there is a need for validation of the PCR techniques before these can be used in a routine laboratory. However, to our knowledge, no study systematically compared the sensitivity of different culture, DNA-extraction, PCR and real-time PCR methods for the detection of *P. aeruginosa *from CF sputum, by using a CF patient sputum based dilution series of *P. aeruginosa*.

Here, we compared the sensitivity of three culture media, five DNA-extraction protocols, two conventional PCR formats and four real-time PCR formats for the detection of *P. aeruginosa*, using a dilution series of *P. aeruginosa *positive sputa in a pool of *P. aeruginosa *negative sputa.

## Results

In this study, we compared the sensitivity of different culture and PCR methods. To that purpose, we prepared a *P. aeruginosa *dilution series in CF sputum by diluting *P. aeruginosa *positive CF patient sputa in a pool of *P. aeruginosa *negative CF patient sputa. This was done instead of diluting cultured *P. aeruginosa *cells in saline or diluting *P. aeruginosa *positive sputum in saline or spiking sputa with *P. aeruginosa *cells, to mimick as closely as possible the sputum samples sent to routine laboratories.

### Comparison of culture methods

No differences in detection limit could be observed between McConjey Agar (MCA) and Cetrimide Agar (CA), i.e. respectively an average of 2 and 3 colonies were counted at dilution eight. For Cetrimide Broth (CB) the detection range was also comparable with that of MCA and CA, i.e. *P. aeruginosa *could be detected up to dilution eight, but the number of colonies was too high to be countable (Table 1).

**Table 1:** Comparison of the sensitivity of different DNA-extraction protocols as assessed by means of conventional PCR combined with agarose gel electrophoresis and by real-time PCR on LightCycler using TaqMan probe

Molecular detection				
**Extraction**	**Protocol**	**Pretreatment**	**Last positive dilution**	

			PCR^a^	Real-time^b^

easyMAG	Generic 2.0.1	Proteinase K	6	8

easyMAG	Generic 2.0.1	None	5	7

easyMAG	Specific B	Proteinase K	5	7

easyMAG	Specific B	None	5	7

High Pure	Manual	Proteinase K	5	6

**Detection by culture**				

McConkey Agar (MCA)			8^c^	

Cetrimide Agar (CA)			8^c^	

Cetrimide Broth with subculture on Blood Agar (CB)			8	

^a ^Conventional PCR with primers PAO1 S and PAO1 A using the Veriti 96-Well Thermal Cycler.

^b ^Real-time PCR with primers PAO1 S and PAO1 A and TaqMan probe *oprL *TM using the LightCycler 1.5.

^c ^The initial inoculum was calculated by averaging the number of cfu at dilution 8 on MC and CA, i.e. 2.5 cfu/50 μl, multiplying with 20 to obtain the cfu/ml, i.e. 50 cfu/ml, multiplying with the dilution factor 1/3125000 to obtain the initial inoculum after dilution with Sputasol, i.e. 78 125 000 cfu/ml, and finally multiplying with factor 2 to obtain the original number of cfu/ml of sputum, i.e. 156 250 000 cfu/ml, or approx. 1.6 log8 cfu/ml.

Based on these results, the number of culturable cells in the original sputum preparation was calculated to be 1.6 log8 cfu/ml.

### Comparison of DNA-extraction protocols

For each sputum dilution, DNA was extracted by four protocols using the bioMérieux easyMAG Nuclisens semi-automated DNA-extractor and by the protocol for the manual High Pure PCR Template Preparation Kit (Roche). Results are listed in Table 1. In our hands, the BioMérieux easyMAG Nuclisens protocol Generic 2.0.1, combined with proteinase K pretreatment, was the DNA-extraction protocol that enabled the most sensitive detection of *P. aeruginosa *from sputum of CF patients, both with conventional and with qualitative PCR, giving amplification of the *P. aeruginosa oprL *target gene up to dilutions 6 and 8, respectively. This DNA-extraction protocol was used further to compare a total of two different conventional PCR and four different (quantitative) real-time PCR formats.

### Comparison of different PCR and real-time PCR formats

Conventional PCR, using the Veriti 96-Well Thermal Cycler (Applied Biosystems), combined with visualisation of the PCR products by agarose gel electrophoresis and ethidium bromide staining respectively by capillary electrophoresis and fluorescence measurement, was compared with three different real-time PCR formats using the LightCycler 1.5 (Roche) and with a commercially available *P. aeruginosa *specific real-time PCR (TaqMan assay) using the ABI7000 (Applied Biosystems). One real-time PCR format used SybrGreen fluorescence as the detection method, whereas the other three real-time PCR formats relied on the fluorescence generated by probes for detection.

Results are listed in Table 2. For the conventional PCR, combined with agarose gel electrophoresis, *P. aeruginosa *DNA could be detected up to dilution 6, while with capillary electrophoresis amplified *P. aeruginosa *DNA could be detected up to dilution 7. *P. aeruginosa *DNA could be detected up to dilution 7 with real-time PCR using SybrGreen, and up to dilution 8 with real-time PCR with the Hybprobes, with the TaqMan probe and with the commercial *Pseudomonas aeruginosa *TaqMan probe detection kit on the ABI7000. In conclusion, the three probe based real-time PCR formats were the most sensitive molecular assays.

**Table 2:** Comparison of the sensitivity of the different PCR formats for sputum dilutions extracted with easyMAG Generic 2.0.1 and proteinase K pretreatment

PCR format^a^	Cycler^c^	Primers	Probes	Annealing temperature (°C)^d^	Last positive dilution
1. PCR + AGE^b^	1	PAO1 S/PAO1 A	None	55	6

2. PCR + FCE	1	PAO1 S/PAO1 A	None	55	7

3. real-time PCR + SybrGreen	2	PAO1 S/PAO1 A	None	55	7

4. real-time PCR + HybProbes	2	*oprL *F/*oprL *R	*oprL*-LC-ROX/*oprL*-LC-FAM	57	8

5. real-time PCR + TaqMan probe^b^	2	PAO1 S/PAO1 A	*oprL *TM	55	8

6. real-time PCR + TaqMan probe	3	Not specified	Not specified	60	8

^a ^AGE: Agarose gel electrophoresis + ethidium bromide staining; FCE: Fluorescent capillary electrophoresis on ABI310.

^b ^PCR formats that were used to compare the sensitivity of the different DNA-extraction protocols (Table 1).

^c ^1: Veriti 96-Well Thermal Cycler, Applied BioSystems, Foster City, Ca.; 2: LightCycler 1.5, Roche, Basel, Switzerland; 3: ABI Prism 7000 Sequence Detection System, Applied BioSystems.

^d ^Annealing temperatures as specified by provider of primers and probes (PCR formats 1-5) or by provider of commercial kit (PCR format 6).

## Discussion

*Pseudomonas aeruginosa *is the major pathogen in cystic fibrosis (CF) patients and is an indicator of poor prognosis in CF patients, especially from the onset of the chronic stage when colonies become mucoid and variant phenotypes emerge. Early detection is essential given the success of early aggressive eradication therapy [6,7]. Therefore, the most prevalent detection and identification methods, i.e. culture and (real-time) PCR, should be optimized to achieve the highest sensitivity.

West *et al*. [21] reported that specific *P. aeruginosa *antibodies were detectable between 6 and 12 months prior to the first positive culture for *P. aeruginosa *from respiratory samples. These findings suggest that culture may miss *P. aeruginosa *in the early stages of colonization. Also at later stages, culture can miss the emerging *P. aeruginosa *phenotypic variants such as the pyoverdine negative mutants, the slowly growing variants, the small colony variants and the auxotrophs, which do not grow on standard media [9,10]. Therefore, the development of improved culture methods and/or of molecular methods is warranted, not only for early detection but also for follow up of colonized patients. However, although several molecular assays for the detection of *Pseudomonas *species have been described (e.g., [11,13-19,22-26]), surprisingly few studies have compared selective and nonselective culture methods with the different molecular methods that have been described for the detection of *P. aeruginosa *directly from clinical samples.

The studies comparing sensitivity of culture and species-specific PCR for the detection of *P. aeruginosa *from sputa of CF patients indicate comparable efficiency of both methods [8,16], with slightly higher sensitivity for PCR in some studies [12,18] or clearly higher sensitivity for PCR [13,26]. We used the PCR format published by De Vos *et al*. [13] in combination with optimized DNA-extraction methods and used in addition real-time PCR to increase PCR sensitivity further. However, using a sputum dilution series of *P. aeruginosa*, and in accordance to most studies, we found no difference in sensitivity between any of the three culture methods and the most sensitive molecular method, i.e. DNA-extraction with easyMAG protocol Generic 2.0.1 and proteinase K pretreatment combined with any of the three probe-based real-time PCRs. In our hands, culture was more sensitive than PCR and SybrGreen based real-time PCR and the difference was even more pronounced when not optimal DNA-extraction methods were used. It should be noticed that we found no difference between selective and nonselective culture methods, but this may be due to the fact that no bacteria, other than *P. aeruginosa *in the two *P. aeruginosa *positive patients, could be cultured from the sputa of the 8 CF patients. As shown in other studies and confirmed here, the pretreatment of the sample and the DNA-extraction protocol strongly influence the sensitivity of the PCR [27,28]. The most sensitive molecular detection method was obtained using the easyMAG Generic 2.0.1 protocol with proteinase K pretreatment in combination with real-time PCR with the TaqMan probe or the HybProbes. Previous studies showed already that the easyMAG extractor is one of the most sensitive and reliable methods for DNA-extraction [29-31]. An additional advantage of automated DNA-extraction like easyMAG might be the lower sample processing variability [28].

Because both approaches, i.e. culture and (real-time) PCR, have important advantages as well as drawbacks [14,20,32,33], in our opinion, both should be or can be combined. PCR technology has the potential to detect the fastidious *P.aeruginosa *variants, which are not detected by the routinely used classical culture procedures [9,10], whereas culture yields a complete genome that can be used for e.g. phenotypic susceptibility testing and whole genome based genotyping techniques like RAPD, PFGE and AFLP [22]. Indeed, several of the published studies indicate that there are instances of culture positive PCR negative samples [11,12,15] as well as culture negative PCR positive samples [11-13,18,19], whereby *P. aeruginosa *infection can only be reliably demonstrated when both approaches are combined.

## Conclusion

In summary, we showed, by testing *P. aeruginosa *positive sputum dilution series, that there is no difference in sensitivity for the detection of *P. aeruginosa *in sputum by selective and non-selective culture and by the most efficient DNA-extraction method combined with the most efficient real-time PCR formats, i.e. the probe-based ones. A prospective study, whereby culture is compared with the DNA-extraction/real-time PCR combination that was established in this study as being the most sensitive, has been started and should learn whether both approaches also yield comparable results when used to detect low inocula of *P. aeruginosa *as can be found after recent infection, in the sputum or nasopharyngeal samples of CF patients not yet colonized by *P. aeruginosa*.

## Methods

### Culture and identification of bacteria

All 8 sputum samples used for this study were collected from cystic fibrosis patients and were cultured on McConkey Agar (MCA) (Becton Dickinson, Cockeysville, MD) and Cetrimide Agar (Cetrimide Broth (Fluka Biochemika, Buchs, Switzerland) + 4% Bacto Agar (Becton Dickinson))(CA) to check for the presence of *Pseudomonas aeruginosa*. The two sputum samples from the chronically infected CF patients yielded only *P. aeruginosa*, as identified by tDNA-PCR and confirmed by OprL PCR [13,34-37], whereas the six sputum samples from the not chronically infected CF patients were culture and PCR negative for *P. aeruginosa*, as tested in the routine laboratory and confirmed by our laboratory.

### Dilution series of *P. aeruginosa *positive sputum in *P. aeruginosa *negative sputum

All 8 sputa were liquefied by adding v/v Sputasol (Oxoid Ltd, Poole, UK) and incubated during 1 hour at 37°C. The two liquefied sputa from the CF patients positive for *P. aeruginosa *were pooled and subsequently diluted tenfold (for dilutions nr 1 and 2) and fivefold (for dilutions nr 3-9) in a pool of liquefied sputa from the six CF patients negative for *P. aeruginosa*. Written informed consent was obtained from the patients for publication of this report. Copies of the written consent are available for review by the Editor-in-Chief of this journal.

### Culture techniques

Fifty μl of each dilution was inoculated onto plates (MCA or CA) or into cetrimide broth and incubated for 24 h at 37°C at ambient atmosphere. Cetrimide Broth was subcultured by inoculating 50 μl onto a Blood Agar plate (Becton Dickinson), which was incubated for 24 h at 37°C (CB). All dilution cultures were done in triplicate and *P. aeruginosa *colonies were counted.

### DNA-extraction protocols

A total of five different DNA-extraction protocols were carried out on each sputum dilution. Two protocols, i.e. Generic 2.0.1. and Specific B, whereby in the latter a double concentration of silica is used and additional washing steps are included, aiming at DNA-extraction from more difficult samples, using the bioMérieux easyMAG Nuclisense extractor (bioMérieux, Marcy-l'Etoile, France), with and without prior proteinase K treatment, were compared with each other and with the manual High Pure PCR Template Preparation Kit (Roche Applied Science, Basel, Switzerland), carried out according to the manufacturer's recommendations. Proteinase K pretreatment consisted of incubation of 200 μl of each sputum dilution during 1 h at 55°C in 200 μl proteinase K buffer (1 mg/ml proteinase K, 0.5% SDS, 20 mM Tris-HCl, pH 8.3) with vortexing every 15 min. For each extraction the start volume was 200 μl of liquefied sputum and the elution volume was 50 μl. Extracted DNA was stored at -20°C prior to PCR.

The quality of the DNA-extracts obtained with the 5 different extraction protocols was compared by conventional PCR, targeting the *oprL *gene with 0.5 μM of primers PAO1 S and PAO1 A in combination with agarose gel electrophoresis and ethidium bromide staining and by *oprL *real-time PCR with 0.5 μM of primers PAO1 S and PAO1 A and 0.1 μM of TaqMan probe *oprL *TM (Table 3).

**Table 3:** Sequences of primers and probes used

Primer/Probe	5'-3' Sequence^d^	Amplicon size (bp)	Reference or source
PAO1 S^a^ PAO1 A^a^	ACC CGA ACG CAG GCT ATG-TET CAG GTC GGA GCT GTC GTA CTC	92	TIB Molbiol

*oprL *F^a^ *oprL *R^a^	ATG GAA ATG CTG AAA TTC GGC CTT CTT CAG CTC GAC GCG ACG	504	[13,28]

*oprL*-LC-FAM^b^	TGC GAT CAC CAC CTT CTA CTT CGA GT-FAM	/	TIB Molbiol

*oprL*-LC-ROX^b^	ROX-CGA CAG CTC CGA CCT GAA G	/	TIB Molbiol

*oprL *TM^c^	FAM-AGAAGGTGGTGATCGCACGCAGA-BBQ	/	TIB Molbiol

^a ^Primers

^b ^HybProbes

^c ^TaqMan Probe.

^d ^TET, FAM and ROX are fluorescent labels. BBQ: BlackBerry quencher

The DNA-extraction protocol, which enabled the most sensitive detection as assessed by these two PCR formats, was used to compare different PCR and real-time PCR formats.

### PCR and real-time PCR formats

Depending on the type of PCR, detection of *P. aeruginosa *was done using two primer sets (Table 2 and Table 3).

Both primer sets are targeting the *oprL *gene because available sequences of different isolates show that this gene is highly conserved http://www.pseudomonas.com/related_links.jsp#alleles. A total of six PCR formats (incl. 4 real-time PCR formats) were compared.

Conventional PCR, using the Veriti 96-Well Thermal Cycler (Applied Biosystems, Foster City, Ca.), was done with primers PAO1 S (TET-labeled) and PAO1 A, whereby PCR products were subsequently visualized either with agarose gel electrophoresis and ethidium bromide staining or with fluorescent capillary electrophoresis. Agarose gel electrophoresis was carried out at 100 V on an agarose gel of 2.5% (w/v), containing 1 mg/ml ethidium bromide and visualized on a UV transilluminator at 540 nm. For capillary electrophoresis, 1 μL of PCR product was added to a mixture of 12 μL deionised formamide, 0.3 μL ROX-labeled GS-400 high-density size standard and 0.2 μL ROX labeled GS-500 size standard. This mixture was then electrophoresed on an ABI PRISM 310 (Applied Biosystems), as described previously [35].

Of the four real-time PCR formats, three were carried out on the LightCycler 1.5 Instrument (Roche) using three different LightCycler real-time PCR kits, all with an optimized MgCl_2 _concentration, i.e. LightCycler FastStart DNA Master^PLUS ^SYBR Green I (Roche), LightCycler FastStart DNA Master^PLUS ^HybProbe (Roche) and LightCycler Taqman Master (Roche) and one was carried out on the ABI7000 instrument, using the commercially available TaqMan *Pseudomonas aeruginosa *detection kit (Applied Biosystems). For all of these PCR formats, the PCR mixes were prepared as recommended by the manufacturer and also the PCR programs were carried out as prescribed by the manufacturer. For the conventional PCRs, the real-time PCR on the LightCycler 1.5 with SYBR Green I and with the TaqMan probe, the annealing temperature was set to 55°C, while for the real-time PCR with the HybProbes the annealing temperature was set to 57°C, as determined by the manufacturer of the primers and probes (TIB Molbiol, Berlin, Germany). For the commercially available TaqMan *Pseudomonas aeruginosa *detection kit the annealing temperature was set to 60°C, according to the manufacturers' instructions.

## Authors' contributions

MV and PD conceived the study. MV, PD, TDB designed the experiments. PD and MV wrote the paper. PD, TDB and LVS performed experiments and analyzed data. JPP, DDV, SVD and FDB helped with the research design and manuscript discussion. SVD and FDB provided patient samples and helped to draft the manuscript. All authors have read and approved the final manuscript.
